# Prevalence and molecular characterization of *Pentatrichomonas hominis* in Siberian tigers (*Panthera tigris altaica*) in northeast China

**DOI:** 10.1111/1749-4877.12629

**Published:** 2022-02-18

**Authors:** Hongbo ZHANG, Nan ZHANG, Pengtao GONG, Shuqin CHENG, Xiaocen WANG, Xin LI, Zhijun HOU, Chang LIU, Tianqi BI, Bobo WANG, Yidan CHENG, Jianhua LI, Xichen ZHANG

**Affiliations:** ^1^ Key Laboratory of Zoonosis Research by Ministry of Education, Institute of Zoonosis, College of Veterinary Medicine Jilin University Changchun China; ^2^ College of Wildlife and Protected Area Northeast Forestry University Harbin China; ^3^ Changchun Animal and Plant Park Changchun China

## Abstract

The overall infection rate of *Pentatrichomonas hominis* in Siberian tigers in northeast China is 31.3%. All the *P. hominis* identified in Siberian tigers belonged to genotype CC1.

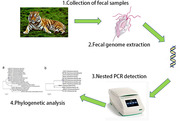

## INTRODUCTION

*Pentatrichomonas hominis*, an anaerobic flagellated protozoan that inhabits the large intestines of mammals and belongs to the Trichomonadidae family (Wenrich 1944; Kim *et al*. 2010; Li *et al*. 2014b, 2016, 2018a, 2020; Maritz *et al*. 2014; Zhang *et al*. 2019), is mainly transmitted through the fecal‐oral route. It was originally presumed to be a commensal protozoan (Tolbert *et al*. 2012) but was found to cause gastrointestinal symptoms, such as diarrhea in humans, dogs, and cats (Gookin *et al*. 2005; Kim *et al*. 2010; Meloni *et al*. 2011; Maritz *et al*. 2014; Bastos *et al*. 2018; Doğan & Tuzemen 2018). It is also associated with systemic lupus erythematosus, irritable bowel syndrome, and rheumatoid arthritis in humans (Jongwutiwes *et al*. 2000; Meloni *et al*. 2011; Compaoré *et al*. 2013). It is well established that approximately 41.54% of *P. hominis* infections are found in Chinese patients with gastrointestinal cancer (Zhang *et al*. 2019). In recent years, awareness of the zoonotic and pathologic potential of *P. hominis* led to the increasing number of studies on the prevalence and pathogenicity of *P. hominis* infections in different vertebrates. *P. hominis* infection has been investigated in humans, domestic animals, and several wildlife species such as sika deer (*Cervus nippon*), rex rabbits (*Oryctolagus cuniculus*), blue foxes (*Alopex lagopus*), silver foxes (*Vulpes vulpes fulva*), raccoon dogs (*Nyctereutes procyonoides*), and minks (*Neovison vison*) (Meloni *et al*. 1993; Inoue *et al*. 2015; Li *et al*. 2015, 2016, 2017, 2018a, 2018b, 2020). However, the prevalence of this parasite in Siberian tigers (*Panthera tigris altaica*) has not yet been assessed.

The Siberian tiger is listed as an endangered species in the world by the International Union for Conservation of Nature, which is included in the CITES Appendix 1 (The Convention on International Trade in Endangered Species of Wild Fauna and Flora) (Guo *et al*. 2014), that only exists in northeast Asia (Tian *et al*. 2014; Peng *et al*. 2016). Approximately 500 wild Siberian tigers survive, and only a small number remains in China accompanying their main activities in the eastern mountain areas of Heilongjiang and Jilin Provinces (Liu *et al*. 2010). As a nationally protected animal in China, Siberian tigers are mainly raised in zoos with enough food supply. Some studies have investigated the pathogenicity of bacteria, viruses, and parasites in Siberian tigers, except for *P. hominis* (Pedersen *et al*. 2007; Moskvina *et al*. 2018). As the infection by *P. hominis* is often found in several wild animals, it is particularly important to check the prevalence of *P. hominis* in the Siberian tiger.

This study aimed to examine the infection and prevalence of *P. hominis* in the Siberian tiger in China. To determine *P. hominis* infection in the captive animal, stool samples of the Siberian tiger were examined by nested polymerase chain reaction (PCR) using partial 18S rRNA and ITS sequences of *P. hominis* as target genes. The finding provide a basis for the prevention and control of the parasite in wild animals. This study is one of the first to focus on *P. hominis* infection and prevalence in the Siberian tiger of China and also provides additional evidence of parasitic infection in this wild animal.

## MATERIALS AND METHODS

### Study population

**Figure 1:**
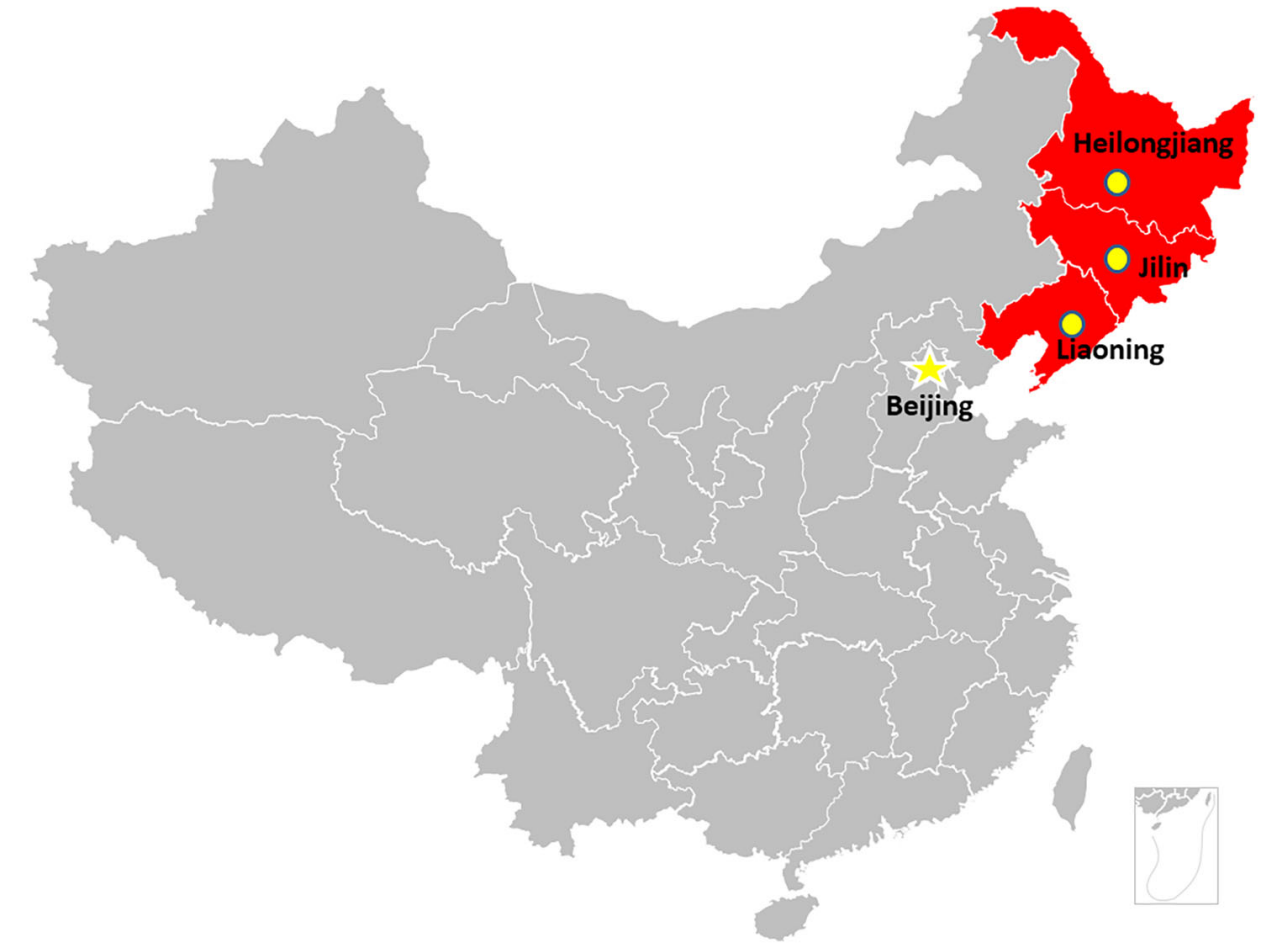
Geographical locations of sample collection sites. The yellow dots indicate the geographical locations at which the samples were collected in this study. The yellow asterisk indicates the location of the capital of China.

The fresh feces of each captive Siberian tiger that were normal (firm but not hard, segmented in appearance) without diarrhoeal symptom was collected and extracted for DNA analysis within 24 h. A total of 131 tiger fecal samples, which is equal to the number of animals was collected from March 2018 to February 2019, and of these, 37 were collected from the Siberian Tiger Garden in Harbin, Heilongjiang Province, 68 from the Animal and Botanical Garden in Changchun, Jilin Province, and 26 from the Siberian Tiger Garden in Shenyang, Liaoning Province (Fig. 1). After releasing the Siberian tiger from the single cage in the morning, the fresh feces in the cage was collected by the breeder, put in a separate self‐sealing bag, labeled, and stored at −20℃. DNA was extracted from the samples within 1 month before PCR analysis. All collection procedures were conducted in strict accordance with the guidelines of the Animal Care and Welfare Committee of Jilin University (IACUC Permit Number: 20160612).

### DNA extraction and PCR analysis

Approximately 200 mg of each sample was used for DNA extraction, and the rest was used for repeated testing. The feces was first homogenized using a homogenizer (Huxi, Shanghai, China), and genomic DNA was extracted from each fecal sample using a QIAamp DNA Stool Mini Kit (Qiagen, CA, USA) according to the manufacturer's instructions. All specimens were analyzed twice. The presence of *P. hominis* in each sample was detected by nested PCR amplifying the partial 18S rRNA gene and ITS sequences as described previously (Kamaruddin *et al*. 2014; Li *et al*. 2016; Table 1). The PCR products were purified using a QIAquick PCR purification kit (Qiagen) and sequenced (Comate Bioscience Co., Ltd., Jilin, China). The work areas for sample preparation, PCR amplification, and sample analysis were strictly separated from each other.

**Table 1:** The information of primers used in this study

Gene	Primer sequence	Reference
18S rRNA	The first primer: F1: ATG GCG AGT GGT GGA ATA R1: CCC AAC TAC GCT AAG GAT T The second primer: F2: TGT AAA CGA TGC CGA CAG AG R2: CAA CAC TGA AGC CAA TGC GAG C	Li *et al*. 2016
ITS	The first primer: F1: CGG TAG GTG AAC CTG CCG TT R1: TGC TTC AGT TCA GCG The second primer: F2: GGT GAA CCT GCC GTT GGA TC R2: TTC AGT TCA GCG GGT CTT CC	Kamaruddin *et al*. 2014

### Sequence alignment and phylogenetic analysis

The 41 sequences obtained were aligned with reference sequences deposited in the GenBank database using BLAST (https://blast.ncbi.nlm.nih.gov/Blast.cgi). Phylogenetic analyses were performed using MEGA7 software (Temple University, Philadelphia, PA, USA) (Kumar *et al*. 2016). The evolutionary distances were calculated by the Kimura 2‐parameter model, and the reliability of cluster formation was evaluated by bootstrapping with 1000 replicates.

### Data analysis

Statistical analysis was performed using SPSS software version 20.0 (IBM Corp., Armonk, NY, USA). The Chi‐square test was used to estimate the statistical significance for fecal samples collected from Siberian tigers of different ages, sexes, regions and seasons. All statistical tests performed were 2‐sided. Odds ratios (ORs) with 95% confidence intervals were calculated to assess the association strengths and were adjusted for both age and sex. Statistical significance was set at *P* < 0.05.

## RESULTS

In total, 41 *P. hominis* infections were identified from 131 fecal samples (31.3%). The infection rate in Heilongjiang province (96.2%, 25/26; χ^2^ = 55.019, df = 1, *P* = 0.001) was the highest, followed by that in Jilin province (22.1%, 15/68; χ^2^ = 6.951, df = 1, *P* = 0.008) and Liaoning province (2.7%, 1/37). The difference in infection rates among the 3 groups was statistically significant (Table 2). Of note, there were also differences in the rates of *P. hominis* infection in different seasons the highest infection rate in Siberian tigers was during winter (100%, 22/22; χ^2^ = 48.107, df = 1, *P* < 0.001), followed by spring (22.06%, 15/68; χ^2^ = 2.690, df = 1, *P* = 0.101) and autumn (9.76%, 4/41). Comparing the infection rate of *P. hominis* in Siberian tigers of different ages, it was found that the infection rate was 88.89% (8/9) in the young (χ^2^ = 14.907, df = 1, *P* < 0.001) and 27.05% (33/122) in the adults. In contrast, there was no significant difference in the infection rate of *P. hominis* between different genders, with the males and females having infection rates of 30.56% (11/36) and 31.58% (30/95) in female, respectively (χ^2^ = 0.013, df = 1, *P* = 0.910; Table 2).

**Table 2:** Occurrence of *P. hominis* infections in Siberian tigers in northeast China

					Logistic regression analysis		
Factor	Category	No. examined	No. positive (%)	χ^2^/df/*P*‐value	% (95% CI)	OR	*P*‐value
Region	Liaoning province	37	1 (2.70%)	–	Reference	1	–
	Jilin province	68	15 (22.06%)	6.951/1/0.008	1.288–80.584	10.189	0.008
	Heilongjiang provnce	26	25 (96.15%)	55.019/1/0.001	53.732–15074.771	900	0.001
Season	Autumn	41	4 (9.76%)	–	Reference	1	–
	Spring	68	15 (22.06%)	2.690/1/0.101	0.804–8.521	2.618	0.101
	Winter	22	22 (100%)	48.107/1/<0.001	4.040–26.003	10.250	<0.001
Age	Adult	122	33 (27.05%)	–	Reference	1	–
	Young	9	8 (88.89%)	14.907/1/<0.001	2.598–179.192	21.576	<0.001
Sex	Male	36	11 (30.56%)	–	Reference	1	–
	Female	95	30 (31.58%)	0.013/1/0.910	0.457–2.407	1.049	0.910
Total		131	41 (31.30%)				

**Figure 2:**
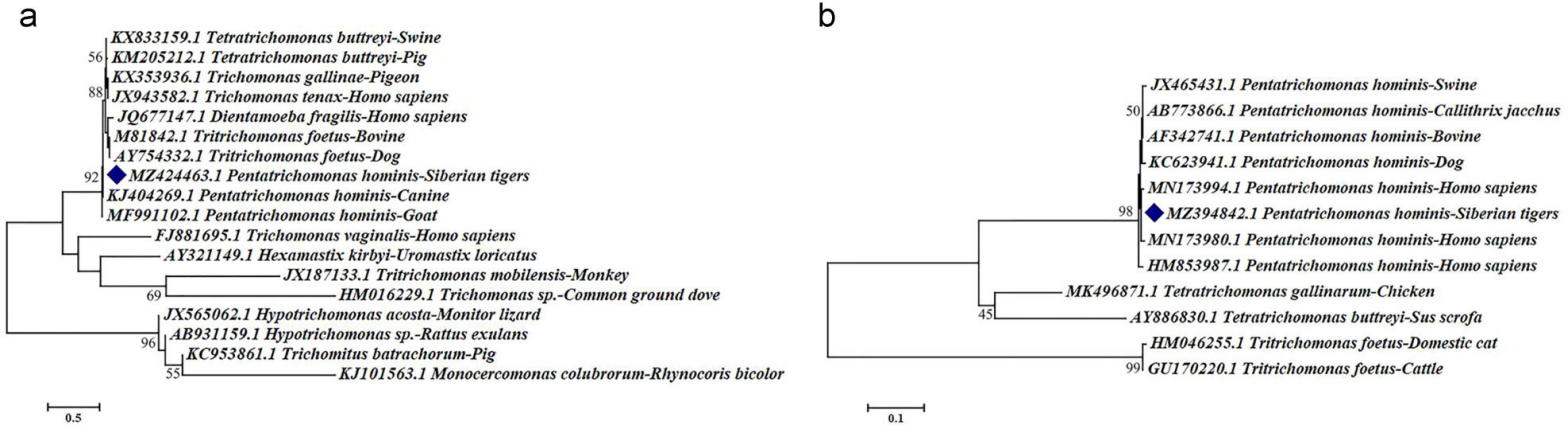
Phylogenetic relationships based on *Pentatrichomonas hominis* 18S rRNA and ITS genes. The phylogenetic relationship between *P. hominis* obtained in this study and other known trichomonads were inferred using the maximum likelihood analysis based on the genetic distance calculated by the Kimura 2‐parameter model. The sequences of *P. hominis* isolated in this study are marked with a diamond. (a) Partial 18S rRNA sequences. (b) ITS sequences.

Molecular characterization of *P. hominis* in Siberian tigers was performed via BLAST analyses of the partial 18S rRNA and ITS sequences. The 18S rRNA sequences (GenBank: MZ424463) obtained had 100% homology with the CC1 genotype (GenBank: KJ408929; Changchun canine strain). Additionally, the ITS sequences (GenBank: MZ394842) obtained were identical to the reference sequence (GenBank: MN173980.1; Changchun human strain), and no SNPs were identified. Finally, phylogenetic analyses demonstrated that all the sequences obtained belonged to *P. hominis* species (Fig. 2).

## DISCUSSION

At present, the occurrences of *P. hominis* have been investigated in wild animals except for humans and livestock (Meloni *et al*. 1993; Inoue *et al*. 2015; Li *et al*. 2015, 2016, 2018a, 2018b, 2020). The highest infection rate was observed in marmosets (*Callithrix jacchus*; 66%), followed by raccoon dogs (*N. procyonoides*; 53.33%) and minks (*N. vison*; 48.33%), indicating high occurrences of *P. hominis* in wildlife (Inoue *et al*. 2015; Li *et al*. 2017). *P. hominis* has been isolated from boas (*Boa constrictor imperator*) and Philippine scops owls (*Otus megalotis*), suggesting its adaptation to different hosts (Dimasuay & Rivera 2013). In addition, it is worth noting that the infection rate of *P. hominis* in laboratory‐bred common marmosets in Japan (66%) is different from that in Siberian tigers reported in this study (Inoue *et al*. 2015), which is lower. As the prey of Siberian tigers in the natural environment, ruminants, such as dairy cattle (6.8%) (Li *et al*. 2020), yellow cattle (4.6%) (Li *et al*. 2020), water buffalo 0.9% (Li *et al*. 2020), and goats (0.3%) (Li *et al*. 2018a), have the potential to infect Siberian tigers with *P. hominis*. The infection rate of *P. hominis* in ruminants was significantly lower than that in Siberian tigers in northeast China. Previous studies have reported that the companion animal can transmit *P. hominis* (Meloni *et al*. 1993; Mostegl *et al*. 2012; Tolbert *et al*. 2012), most of the companion animals are infected with *P. hominis* with diarrhea‐like symptoms (Gookin *et al*. 2005; Kim *et al*. 2010; Bastos *et al*. 2018). *P. hominis* infection in companion animals has been reported worldwide. For example, cats are be infected with *P. hominis* in Austria (0.98%) (Mostegl *et al*. 2012), Brazil (3.89%) (Santos *et al*. 2015), and Thailand (20.25%) (Mahittikorn *et al*. 2021), and the infection rate of *P. hominis* in kittens in Japan is 0.5% (Itoh *et al*. 2020). Dogs can also be infected by *P. hominis* in the United States (92.85%) (Tolbert *et al*. 2012) and Poland (12.19%) (Michalczyk *et al*. 2015), and the infection rate of *P. hominis* in puppies in France is 15.8% (Grellet *et al*. 2013). The infection rate is related to many factors, such as examination method, age, sample size, and season, among others.

In the present study, we detected the occurrences of *P. hominis* in Siberian tigers in northeast China. The infection rate of *P. hominis* in Siberian tigers was 31.3%. Occurrences were significantly different among Heilongjiang (96.2%), Jilin (22.1%), and Liaoning provinces (2.7%). The different infection rates of *P. hominis* in Siberian tigers may be due to the following reasons: 1) natural infection with *P. hominis*; 2) transmission by their mother or a polluted environment; 3) transmission via exchanges of Siberian tigers, which may be infected by *P. hominis*, between different zoos. Also, it may be due to predation of other wild animals such as wild rats that may act as reservoirs of *P. hominis* for Siberian tigers in the environment, in which rats are naturally infected with *P. hominis* (Fukushima *et al*. 1990; Grellet *et al*. 2013). These require further exploration. To date, there are few reports on the pathogenicity of *P. hominis* infection in humans and animals (Meloni *et al*. 2011; Kamaruddin *et al*. 2014; * *Doğan & Tüzemen 2018). Some animals, such as dogs and cats, may have diarrhea, but most do not exhibit obvious symptoms of diarrhea (Gookin *et al*. 2005; Li *et al*. 2014a; Bastos *et al*. 2018). In this study, a similar situation was observed in which fecal samples of Siberian tigers infected by *P. hominis* was normal (firm but not hard, segmented in appearance) without diarrheal symptoms. Further studies are needed to confirm the pathogenicity of *P. hominis* infection in Siberian tigers.

To date, the most common subtype of *P. hominis* is the CC1 (Changchun Canine 1) genotype, which has been identified in humans (GenBank: KJ408960 and MK177542), dogs (GenBank: KJ408929 and KJ404269), cats (GenBank: MG015711), monkeys (GenBank: KJ408932), goats (GenBank: MF991102), and wildlife (Li *et al*. 2016, 2017, 2018a). Genetic analysis of the 18S rRNA sequences revealed that all *P. hominis* obtained in this study belonged to the genotype CC1, suggesting potential zoonotic transmission of *P. hominis* between Siberian tigers and other hosts. Interestingly, alignment of the ITS sequences indicated that they were homologous to the reference sequence MN173980.1 isolated from patients with cancer (Zhang *et al*. 2019). Therefore, Siberian tigers can act as a natural host for *P. hominis* and also as a transmission source for *P. hominis* infections in humans (Maritz *et al*. 2014). Lastly, phylogenetic analysis revealed that the 18S rRNA and ITS sequences were genetically clustered with other known *P. hominis* sequences, further supporting potential zoonotic transmission. In this study, we found that *P. hominis* infection in Siberian tigers occurs in northeast China, providing additional data on the parasitic infection of Siberian tigers. It provides an important basis for the control of parasitic infection in Siberian tigers and promoting the health of captive wild animals.

This study is the first to report *P. hominis* infection in Siberian tigers that belongs to the CC1 genotype, whichis found in humans, dogs, and other wild animals. However, the effects of *P. hominis* infection in Siberian tigers should be further investigated.

## CONFLICT OF INTEREST

The authors declare no conflict of interest.
